# Gun owners’ assessment of gun safety policy: their underlying principles and detailed opinions

**DOI:** 10.1186/s40621-023-00430-z

**Published:** 2023-04-17

**Authors:** Kathleen Grene, Amani Dharani, Michael Siegel

**Affiliations:** Department of Public Health and Community Medicine, Tufts University School of Medicine, 136 Harrison Avenue, Boston, MA 02111 USA

**Keywords:** Firearms, Firearm laws, Firearm violence, Gun policy, Guns, Survey study

## Abstract

**Background:**

While gun owners are frequently surveyed, we are not aware of any study that has examined principles held by gun owners that underlie their gun policy opinions, or their opinions about specific provisions of each policy. To find the common ground between gun owners and non-gun owners, this paper aims to answer the following: (1) What underlying principles affects gun owner support for gun policies; (2) how do gun owners’ attitudes change depending on the specific provisions within these policies?

**Methods:**

In May 2022, a survey was administered by NORC at the University of Chicago and completed by adult gun owners (*n* = 1078) online or by phone. Statistical analyses were performed using STATA. The survey used a 5-point Likert scale to evaluate gun owners’ principles and attitudes toward firearm regulation, such as red flag laws, and possible provisions to these policies. Focus groups and interviews were conducted with 96 adult gun owners and non-gun owners to further clarify points in the survey for the former and to ascertain support for the same policies and their potential provisions for the latter.

**Results:**

The principle that gun owners identified with the most concerned keeping guns out of the hands of those with an increased risk for violence. There was significant overlap among gun owners and non-gun owners on policy support, especially with this central theme that those with a history of violence should be prevented from accessing a firearm. The degree of support for policies was different depending on what provisions were said to be included in the policy. For example, the degree of support for universal background checks ranged from 19.9 to 78.4% depending on the details of the legislation.

**Conclusion:**

This research demonstrates common ground between gun owners and non-gun owners: It informs the gun safety policy community about gun owners’ views and principles on gun safety policy and which policy provisions impact their support for a given law. This paper suggests that an effective, mutually agreed upon gun safety policy is possible.

**Supplementary Information:**

The online version contains supplementary material available at 10.1186/s40621-023-00430-z.

## Background

Gun violence continues to surge in the USA, with over 45,000 people dying in 2020 as a result of a firearm (CDC). Gun owners and non-gun owners are often portrayed as holding conflicting views on the root causes of, and solutions to, gun violence, with gun owners unlikely to support gun violence prevention policies that non-gun owners see as important to implement. Despite staunch opposition by gun rights advocacy groups, surveys reveal that the majority of gun owners support the most common firearm policies (Bloomberg 2018, Gallup 2017). This suggests that there are shared underlying values and principles between gun owners and non-gun owners. Although several previous studies have assessed attitudes of gun owners toward specific gun policies, we are not aware of any survey that has examined the values and principles held by gun owners that underlie their opinions about these policies. Moreover, previous studies have ascertained gun owner attitudes toward policies with a simple “yes” or “no” question; we are not aware of any that have investigated opinions about the detailed provisions in these laws. This paper aims to answer two critical questions: (1) What underlying principles affect gun owner support for gun policies; and (2) how do gun owners’ attitudes change depending on the specific provisions within these policies?

Existing research has focused primarily on gun owners’ general opinions regarding gun policies, such as research conducted at Johns Hopkins where researchers have found that gun owners’ support a broad range of gun violence prevention laws (Barry, Crifasi). Most recently, a study by Crifasi et al. (2021) examined support for gun policies by race and ethnicity among gun owning subgroups. It is concluded that most American adults supported 17 of 21 gun-related policies and specified support among gun owners by race and ethnicity. Another study published by Kruis et al. (2021) assessed characteristics of gun owners and their perceptions of various gun control policies. A 2020 opinion survey explored the difference in private support versus publicly stated support of gun policy among US adults (Dixon et al. 2020). It is concluded that many US adults failed to recognize that most gun owners support key gun safety policies and that many gun owners are unwilling to publicly share their individual support for certain gun policies (Dixon et al. 2020). A number of earlier studies have examined gun owner attitudes toward various gun policies (e.g., Barry 2019, Bloomberg 2018, Merrill-Francis et al. 2021, Shepperd 2018, Stone et al. 2017, Wintemute 2014, Ye 2022). However, these studies have two major limitations which inhibit our ability to craft a policy platform that gun owners will support.

First, while there have been numerous surveys to measure gun owner opinions toward specific policies, these surveys have not explored the values and principles that drive these opinions. For example, we know that 86% percent of gun owners support universal background checks (Gallup 2017) but not the reasons underlying this support. What criteria do gun owners believe should disqualify someone from purchasing or possessing a gun? Should a background check be required when a family member is purchasing or borrowing a gun? Should background checks be used to create a gun registry? Similarly, we know that 77% percent of gun owners support a permit requirement for the purchase of a handgun (Siegel and Boine 2019); however, this tells us nothing about what the criteria should be for issuing such a permit. The answers to these questions are critical for public health practitioners or policy makers in crafting a platform of gun policies that gun owners will support.

Second, previous surveys have not examined the nuances of gun owner support for policies; in other words, the specific provisions would need to be present in a proposed law in order to garner public gun owner support. Although we know that 86% percent of gun owners generally support background checks (Gallup 2017), would they still support such a law even if it prevented them from lending guns to family members for a short period of time without going through a cumbersome background check process at a federally licensed gun dealer? Although 81% percent of gun owners support red flag laws (Siegel and Boine 2019), would they still support such a law even if it failed to include a provision that imposes a fine on people who make a false or vindictive claim that a gun owner is a danger? No prior survey has obtained this level of detail regarding gun owner support for gun policies.

By (1) identifying the underlying principles that drive gun owners’ support or opposition to firearm policies; and (2) understanding gun owner opinion about policies down to the level of their specific detailed provisions, this research could help gun violence prevention advocates and policy makers build a comprehensive package of gun policies that has a high likelihood of support from both gun owners and non-gun owners. Ultimately, this research aims to inform the development of a gun policy platform that provides a “win” for both sides and achieves the dual objectives of promoting effective gun policy reform without alienating gun owners.

## Methods

### Survey design

The primary purpose of the survey was to identify the common ground between gun owners and non-gun owners that could serve as a starting point for the development of effective gun legislation that would have a high likelihood of being supported by both sides. Second, the survey aimed to identify the specific provisions of gun legislation that most concern gun owners. During the development of the survey, we consulted with many gun owners and with the leadership of gun rights’ organizations. There were two major sections of the survey: (1) evaluating gun owners’ principles as they apply to gun regulation; and (2) gaining feedback on gun owners’ attitudes toward specific provisions of gun laws. The survey instrument is included as Additional file 1.

### Survey administration

To ascertain the attitudes and opinions of gun owners, we conducted a national internet-based and phone-based survey of 1078 adult gun owners. The survey was conducted by NORC at the University of Chicago (NORC). NORC is one of the few survey companies that maintains an internet panel that was recruited using probability sampling techniques. Unlike most panels, which are opt-in, NORC knows the selection probability for each panelist and is therefore able to generate weights that allow for nationally represented estimates to be made. The survey respondents were adult, gun-owning members of the NORC AmeriSpeak panel, a pre-recruited internet panel of approximately 50,000 adults throughout the USA who have agreed to take periodic internet surveys.

### Sampling procedure and response rate

A total of 11,101 adult members of the AmeriSpeak panel were invited by e-mail to complete a screening to determine whether they were eligible to take the survey. Respondents who reported that they owned a gun were deemed eligible and were invited to take the survey online through the password-protected AmeriSpeak Mobile App, the password-protected AmeriSpeak Web portal, or by following a link in the e-mail invitation sent to them.

To encourage study cooperation, NORC sent the initial invitation and email reminders to sampled web-mode panelists on the following dates: (1) initial invitation: Tuesday, May 10, 2022; (2) first email reminder: Saturday, May 14, 2022; and (3) second email reminder: Tuesday, May 17, 2022. To administer the phone survey, NORC dialed sampled panelists who prefer to take surveys on the phone from May 11 to May 18, 2022. Although most panelists who have stated a preference to take the survey on the phone do take them in that mode, they also have the option of taking the survey online via the web portal or the AmeriSpeak App or can ask the interviewer to email them a survey link instead. Panelists were offered 5000 AmeriPoints, worth the cash equivalent of $5.00, for completing this survey.

Of the 11,101 invited panelists, 2656 completed the screener questions to determine their eligibility for the survey. Of these, 1137 were determined to be eligible for the survey. Of these eligible panelists, a total of 1078 completed the survey. The screener completion rate was therefore 23.9%, and the survey completion rate was 94.8%. This resulted in an overall survey completion rate (response rate) of 22.7%.

### Weighting procedure

The data were statistically weighted by NORC to account for the following factors: (1) the initial probability of panel member selection into the panel; (2) panel recruitment non-response: (3) post-stratification of the recruited panel to match population benchmarks; (4) selection probabilities for the study sample; and (5) survey non-response. NORC provided the researchers with study-specific final weights that were applied in all analyses to generate estimates that apply to the national population of gun owners.

### Survey measures

The first section of the survey evaluated the principles that gun owners’ have in relation to regulation. Respondents were asked to indicate their support for a series of gun policy principles by stating whether they strongly agreed, agreed, disagreed, strongly disagreed or felt neutral. Examples of principles we inquired about were: (1) People who have been convicted of a violent crime should not be able to purchase or possess a gun; (2) owning a gun for self-defense is a fundamental constitutional right; and (3) one of the primary goals of gun laws should be to keep guns out of the hands of people who are at high risk of violence, while allowing law-abiding citizens to obtain guns.

The second section of the survey evaluated gun owners’ attitudes to specific gun law provisions. Respondents were asked about four different policy provisions that fell under the buckets of background checks, red flag laws, conceal carry permits, and permits to possess any gun and indicated whether they strongly agreed, agreed, disagreed, strongly disagreed or felt neutral about each statement. For example, for universal background checks, we asked respondents to indicate their level of agreement with the following statements: (1) I would only support a universal background check law if it included an exemption to allow the sale and transfer of guns to a family member without a background check; (2) I would only support a universal background check law that did NOT exempt sales to family members if private sellers were given privacy-protected access to the National Instant Criminal Background Check System (NICS) database, whereby a green light or red light would appear to indicate whether the person is eligible; (3) I would only support a universal background check law if it provided a way to sell or transfer firearms to family members without having to go through a federally licensed dealer (FFL).

Through these questions we divided gun owners into three categories for each of the four policies; using universal background checks as an example: (1) those who would support universal background check laws regardless of the specific provisions; (2) those who would oppose universal background check laws regardless of the specific provisions; and (3) those whose support for universal background check laws would depend on the specific provisions. Within the third category, we were able to determine which law provisions were most important to garner gun owner support.

A 5-point Likert scale was used for all opinion questions. We defined support for a principle or a policy provision as being responses of strongly agree and agree (strongly support and support), with the other three responses classified as not supporting that principle or policy.

### Survey data analysis

All statistical analyses were carried out using STATA version 17 (StataCorp, College Station, TX). Likert scale was used in the survey, but we collapsed this in our results because we found that a dichotomous variable facilitated interpretation.

### Post-survey focus group design and implementation

We conducted a series of post-survey focus groups and interviews. We did not have the resources to survey non-gun owners in addition to gun owners without sacrificing our ability to derive relatively precise estimates for gun owners, especially sub-groups. We therefore sought to conduct qualitative, post-survey research in which we directly interviewed a sample of non-gun owners to confirm that they agreed with the “common ground” principles identified in the survey. We also sought to ascertain the opinions of leaders of major national gun violence prevention organizations toward these guiding principles.

Focus groups and interviews were performed by reaching out to a variety of organizations. There were a total of 96 participants. Gun owners and non-gun owners were included in this phase. Focus groups and interviews were conducted over Zoom and took 45 min to 1 h and 30 min. Focus groups had two to six participants, and a number of one-on-one interviews were performed as well. Participants were sent a $10 Amazon gift card via email to thank them for their time.

## Results

### Survey results

#### Sample representativeness

Of the 1078 gun owners in our survey sample, a small majority were male, the age group 60 + years had the most respondents, the majority were white, and Republicans made up the greatest percentage for political party affiliation (Table 1). The demographic findings resemble national gun owner statistics, such as the National Firearms Survey (NFS) of 2021, which surveyed 15,450 gun owners (English 2021). For example, the NFS of 2021 found that 57.8% of gun owners are males, and 42.2% are females; similarly, our survey found that 55.5% are males, and 44.5% are females. Race demographics were also similar: For example, 9.0% of our survey respondents were Black, compared to the NFS’s survey where 10.6% of participants identified as Black. The similarity in demographics between our survey’s respondents and the NFS’s survey respondents, and our sample size, provides evidence that our survey sample is a reasonably representative sample of US gun owners.

**Table 1:** Demographics

	Percentage	95% Confidence interval
*Gender*		
Male	55.5	(51.8–59.2)
Female	44.5	(40.8–48.2)
*Age (years)*		
18–29	16.0	(13.2–19.3)
30–44	26.7	(23.7–30.0)
45–59	25.0	(21.9–28.4)
60 +	32.3	(28.9–35.8)
*Race*		
White, non-Hispanic	70.6	(67.0–74.0)
Black, non-Hispanic	9.0	(7.1–11.3)
Other, non-Hispanic	1.5	(0.8–2.9)
Hispanic	14.3	(11.7–17.3)
2 + , non-Hispanic	3.1	(2.1–4.6)
Asian, non-Hispanic	1.5	(0.7–3.3)
*Political party affiliation*		
Democrat	32.6	(29.2–36.1)
Independent	17.9	(15.1–21.1
Republican	49.6	(45.8–53.3)

#### Principles underlying gun owners’ opinion on gun safety policies

The principles that gun owners identified with the most concerned keeping guns out of the hands of those with an increased risk for violence, while permitting gun ownership for law abiding citizens (Table 2). For example, 80.7% of gun owners agreed with the statement “People convicted of a violent crime should not be able to purchase or possess a gun.” When analyzing this principle by gender, both male and female respondents agreed with it to a similar degree; support for this principle increased with age, and Democrats and Republicans supported it to a similar degree as well. When it comes to the Second Amendment, 82.9% believe that owning a gun for self-defense is a Constitutional right, but only 36.5% think that this right is “absolute” and cannot be infringed upon under any circumstances. Most gun owners (77.0%) believe that their Second Amendment right is protected, but that there are exceptions. The majority of gun owners are concerned about gun violence in the country, from homicides and gun crimes in cities to mass and school shootings. Furthermore, gun owners want to help find a way to reduce gun deaths and injuries (70.1%). However, it is important to recognize that this also means that there is a smaller subgroup of gun owners who take a hardline stance on the Second Amendment, do not view gun violence as a problem, and have no interest in being involved in efforts to reduce gun deaths.

**Table 2:** Principles underlying gun owners’ opinion on gun safety policies

Principle	Overall (%)	Gender		Age group (years)				Political affiliation		
		Male (%)	Female (%)	18–29 (%)	30–44 (%)	45–59 (%)	60 + (%)	Democrat (%)	Independent (%)	Republican (%)
People convicted of a violent crime should not be able to purchase or possess a gun	80.7 (77.6–83.6)	81.0	79.3	72.6	78.2	80.8	86.9	82.4	70.6	83.3
One of the primary goals of gun laws should be to keep guns out of the hands of people who are at high risk of violence, while allowing law-abiding citizens to obtain guns	80.5 (77.2–83.3)	79.0	82.3	67.4	81.3	77.5	88.6	86.0	67.7	81.4
Owning a gun for self-defense is a fundamental constitutional right	82.9 (79.7–85.6)	85.8	79.2	68.4	83.6	86.6	86.5	71.2	69.1	95.3
The right to own a gun is absolute and cannot be altered in any way	36.5 (32.9–40.2)	41.4	30.4	32.8	32.9	40.4	38.3	22.3	31.6	47.5
Like free speech, the right to own a gun is constitutionally protected, but there are exceptions to this right	77 (73.6–80.1)	79.0	74.4	67.7	72.0	79.7	83.7	78.0	60.5	82.3
I am concerned about the increase in gun-related homicides and gun crimes in cities	65.5 (61.7–69.1)	63.8	67.5	49.6	54.3	66.0	82.4	79.8	49.2	61.7
I am concerned about the frequency of mass shootings	71.2 (67.5–74.6)	68.2	74.9	53.6	67.0	71.9	83.0	88.3	60.3	63.7
I am concerned about the frequency of school shootings	74.6 (71.0–78.0)	72.7	77.1	54.4	71.8	75.6	86.3	89.7	66.1	67.8
I want to help find a way to reduce gun deaths and injuries	70.1 (66.3–73.6)	67.7	73.1	56.8	69.0	71.2	76.8	85.4	58.4	64.1

#### Initial policy opinions

Policy support is provision-dependent for the majority of respondents (Table 3). Gun owners are not immediately opposed to policies like universal background checks and red flag laws, but are more concerned with specific provisions. For example, only 19.9% of respondents unconditionally support universal background checks, which at first glance can appear as though the minority of gun owners support such a policy. However, 62.9% of respondent support for universal background checks is provisional. In other words, it is not that the minority of gun owners are in favor of universal background checks; rather, the majority of gun owners support these checks, contingent upon the details of such a law. We noticed similar findings for the remaining three laws: red flag laws, permits to purchase or possess any gun and concealed carry permits. These laws, and the provisions that gun owners would or would not like to see in them, are examined in Tables 4, 5, 6 and 7.

**Table 3:** Policy opinions

Law	General policy opinion^a^ (%)	Unconditionally support (%)	Unconditionally oppose (%)	Provision dependent (%)
Universal background checks	72.9	19.9	17.3	62.9
Red flag laws	69.2	17.3	14.2	68.2
Permit to purchase or possess any gun	47.5	16.6	20.1	63.3
Concealed carry permits laws	63.9	22.3	13.8	63.9

^a^Policy opinion when asked general question about a given policy

**Table 4:** Percentage of gun owners who would only support background checks if specific provisions were present

Opinion	Agree (%)	95% Confidence interval
I would only support a universal background check law if it included an exemption to allow the sale and transfer of guns to a family member without a background check	21.1	(18.0–24.5)
I would only support a universal background check law that did NOT exempt sales to family members if private sellers were given privacy-protected access to the NICS database, whereby a green light or red light would appear to indicate whether the person is eligible	17.0	(14.3–20.0)
I would only support a universal background check law if it provided a way to sell or transfer firearms to family members without having to go through a federally licensed dealer (FFL)	25.0	(21.8–28.6)
I would only support a universal background check law if it did not result in the creation of a firearm registry (i.e., if a permanent record was kept of the name of the buyer and the type/model of guns purchased)	21.5	(18.5–24.9)
I would only support a universal background check law if it required a timely response from the NICS check system, such as within 72 h	25.7	(22.5–29.1)

**Table 5:** Percentage of gun owners who would only support red flag laws if specific provisions were present

Opinion	Agree (%)	95% Confidence interval
I would only support a red flag law if it included a provision stating that the firearm could only be confiscated for an extended period of time after a timely due process hearing in front of a judge, at which point the subject of the potential order could be present and provide evidence	36.2	(32.7–39.9)
I would only support a red flag law if it included a provision stating that the request to remove the firearm from the person could only be made by a law enforcement officer, not a family member	18.4	(15.6–21.5)
I would only support a red flag law if it included a fine for anyone who dishonestly uses the law to try to get firearms taken away from another person (for example, the person filing the complaint is just trying to get back at the gun owner for some reason)	43.7	(40.0–47.5)
I would only support a red flag law if it included a protocol for expeditious and inexpensive restoration of Second Amendment rights if the accusation proves to be unfounded or when the person is deemed to no longer represent a threat	39.3	(35.7–43.0)
I would only support a red flag law if it included provisions for the expeditious return of the accused person’s firearms once the order is lifted and the accused person’s rights are restored	41.6	(37.9–45.3)
I would only support a red flag law if it allowed the transfer of confiscated firearms to a designated friend or family member for safekeeping, instead of being stored by law enforcement officials	22.5	(19.5–25.8)

**Table 6:** Percentage of gun owners who would only support permits to purchase or possess a gun if specific provisions were present

Opinion	Agree (%)	95% Confidence interval
I would only support a permit to purchase law if it required live firearm shooting training to obtain the license or permit	24.6	(21.5–28.0)
I would only support a permit to purchase law if it exempted gun owners who have a permit from the need for a background check when purchasing or borrowing a gun from a family member	19.3	(16.5–22.5)
I would only support a permit to purchase law if it exempted gun owners who have a permit from the need for a background check when purchasing any new gun	22.0	(19.0–25.4)
I would only support a permit to purchase law if it could not be used to create a registry of firearms owned by each gun owner (i.e., the name of the person getting the license would be linked to the number and type of firearms that they purchase)	18.9	(16.1–22.0)
I would only support a permit to purchase law if it included perks associated with the permit, such as waiving of the normal waiting period for purchase of a gun	15.5	(12.9–18.5)
I would only support a permit to purchase law if the permit were available at low cost	24.7	(21.6–28.1)
I would only support a permit to purchase law if it allowed the permitting process to be completed online	13.5	(11.0–16.4)

**Table 7:** Percentage of gun owners who would only support concealed carry permit laws if specific provisions were present

Opinion	Agree (%)	95% Confidence interval
I would only support a concealed carry permit law if it required live firearm shooting training as part of the procedure to obtain the permit	34.0	(30.6–37.6)
I would only support a concealed carry permit law if it could not be used to create a registry of firearms owned by each gun owner (i.e., the name of the person getting the license would be linked to the number of types of handguns that they own)	22.6	(19.6–26.0)
I would only support a concealed carry permit law if it was “shall issue,” meaning that law enforcement officials would have no discretion to deny a permit if the applicant met all the specified criteria, such as not having a history of a violent crime	28.2	(24.9–31.7)
I would only support a concealed carry permit law if it included perks associated with the carry permit, such as waiving of the normal waiting period for purchase of a gun	18.1	(15.4–21.3)
I would only support a concealed carry permit law if the permit was available at low cost	23.6	(20.6–26.9)
I would only support a concealed carry permit law if it allowed the application procedure to be completed online	10.5	(8.3–13.1)

#### Provisional support for universal background checks

Of all provisions listed, the ones that were most important for gun owners to see in a universal background check law were a way to sell or transfer firearms to family members without going through an FFL and a timely response from the NICS system (Table 4). For these two provisions, 25.0% and 25.7% of gun owners indicated that their support for a universal background check law is contingent upon including these provisions. The third most important provision—not creating a firearm registry—was demanded by 21.5% of gun owners in order to support a universal background check law. Only 19.9% of gun owners unconditionally supported universal background checks, and 68.2% of respondents support for such a law was provision-dependent, but 78.4% (75.3–81.2% CI) of gun owners supported the law if all these provisions were included (data not shown).

We found that gun owners are sensitive to whether background checks would result in a registry (data not shown). Of those who always oppose background checks (237 respondents) the vast majority (205 or 86.5%) believe that universal background checks would result in a registry (Pr = 0.000). 17% of respondents believe a background check would not result in a registry, but if it did, would not support it.

#### Provisional support for red flag laws

For red flag laws, there are four provisions that gun owners would like to see in order to support the policy: (1) A firearm can only be confiscated for an extended period of time *after* a timely due process hearing from a judge where the subject of the potential order can present and provide evidence; (2) a fine for anyone who dishonestly uses the law, i.e., vindictive use; (3) a protocol for expeditious and inexpensive restoration of Second Amendment rights if the accusation proves to be unfounded or the person is no longer deemed a threat; (4) expeditious return of firearms once the order is lifted and the accused’s gun rights are restored (Table 5). Of these four provisions, the one that survey respondents found the most essential for a red flag law is a fine for anyone who dishonestly uses the law: 43.7% agreed that they would only support the law if this provision were included. This suggests that failure to include such a provision, and failure to guarantee expeditious return, would result in loss of majority support among gun owners. Just 17.3% of gun owners supported red flag laws under any conditions, 68.2% of respondents support for red flag laws was provision-dependent, and 81.0% (77.8–83.8% CI) of gun owners supported the law if all provisions were included (data not shown).

#### Provisional support for permits to purchase or possess any gun

Of all provisions listed, the ones that were most important for gun owners to see in a permit to purchase or possess any gun law were a requirement to first complete live firearm shooting training, and for the permit to be available at a low cost (Table 6). For these two provisions, 24.6% and 24.7%, respectively, of gun owners would only support this policy on the grounds that they were included. Only 15.5% of respondents support for such a law was contingent upon the inclusion of perks in the law, such as waiving the normal waiting period for the purchase of a gun. Just 16.6% of gun owners supported permits to purchase or possess a gun under any conditions, 63.3% of respondents said their support was provision-dependent, and 75.5% (72.2–78.6% CI) of gun owners supported the law if all provisions were included (data not shown).

#### Provisional support for concealed carry permit laws

Of all provisions respondents were presented with, the one that stood out as most important for gun owners to see in a concealed carry permit law was a requirement to first complete live firearm shooting training; 34.0% of gun owners stated that they would only support a concealed carry permit law if this provision were included (Table 7). A total of 22.6% of survey respondents needed to see a provision explicitly stating that a concealed carry permit law cannot result in a registry in order to support it. Similar to the law regarding a permit to purchase or possess any gun, 18.1% of survey respondents said that a provision that required perks with the concealed carry permit was necessary in order to support it. The provision that was considered the least important to respondents was a requirement that the application can be completed online (10.5% of gun owners stated that this provision was mandatory for their support of the law). 22.3% of gun owners unconditionally supported concealed carry permit laws, 63.9% of respondent support was provision-dependent, and 81.0% (77.8–83.8% CI) of gun owners supported the law if all provisions were included (data not shown).

#### Focus group results

There was broad support among gun owners and non-gun owners for the principle that people with a history of violence should not be able to access firearms and for policies that implemented this principle by preventing violent criminals (even at the misdemeanant level) from purchasing or possessing guns. The details of this concept varied, but the only individuals who opposed this idea were those who felt that criminals should not be released from prison until we are prepared to reinstate all constitutional rights upon prison release. The views of those largely opposed to gun safety policy had a smooth overlap with those in favor of certain new gun safety policies because the former group applied “tough on crime” rhetoric, stating that criminals should lose their gun ownership and purchasing rights for some amount of time.

## Discussion

To the best of our knowledge, this is the first paper to ascertain the principles that underlie gun owners’ views toward gun safety policy, and to assess gun owners’ attitudes and opinions for specific provisions in gun laws. We found that the primary principle upon which gun owners base their opinions about gun policy was that such policies should ensure that people who are at high risk for violence cannot access guns, while interfering minimally with the ability of law-abiding gun owners to purchase and possess guns. We also discovered that gun owners are sensitive to specific provisions in these laws, to the extent of finding that anywhere between 16.6 and 82.4% support a given gun safety policy depending on the provisions included. The combination of law-abiding gun owners’ views on keeping guns away from violent criminals and their freedom to exercise their Constitutional rights suggests that gun owners want policies that reduce crime, but they also want their own rights to gun possession and purchasing to be guaranteed.

Attitudes toward the four policies covered in detail in the survey reveal that gun owners support for each one increases depending on which provisions are included. Universal background checks would need to include a way to sell or transfer firearms to family members without going through an FFL and be able to ensure timely response through NICS. It appears that opinions on background checks are in part influenced by whether it would result in a gun registry. Our findings could mean that those who oppose background checks oppose them, at least in part, because they believe that checks would result in a registry. Regardless, if the provisions included here are in a universal background check law, gun owner support for such a law could increase from 72.9 to 78.4%. For red flag laws, inclusion of the six provisions here could increase gun owner support for them by over 10%, from 69.2 to 81.0%. The inclusion of seven provisions to a permit to purchase or possess a firearm policy vastly increases gun owner support, by 28 percentage points, from 47.5 to 75.5%. Lastly, a law for concealed carry permits increases, from 63.9 to 82.4%, when the six provisions here are incorporated into such a law.

### Research implications

Our findings suggest that all previous research asking general policy questions with binary “yes/no” responses has not fully assessed gun owner opinion. We have learned that questions that assess granular provisions for each gun safety law are required to get the true perception of gun owner opinion. Future surveys of gun owners’ public opinion should assess specific aspects of laws to know where gun owners stand.

### Practice implications

This research shows that it is important to talk to gun owners and get their input when crafting these policies. There are specific provisions to gun safety policy that, if included, may increase support. For example, a law requiring permits to purchase any firearm initially appears not to have much support among gun owners. However, upon closer inspection, the inclusion of specific provisions or assurances—such as ensuring that a permit is available at low cost, including safety training as part of the permitting process, and creating an exemption that would allow family members to sell or loan a firearm to a family member without having to complete a background check whether the purchaser and/or borrower possesses a valid firearm permit—drastically changes support.

### Policy implications

The most important finding of our research was that gun owners believe that people who are at a high risk for violence should not be able to access firearms, but that law abiding gun owners should be minimally inconvenienced. Several policy options are consistent with this principle. First and foremost would be a law that prohibits anyone who has committed a violent crime from purchasing a firearm. This would fulfill the principle of keeping guns out of the hands of people at a high risk of violence while not interfering at all with the ability of law-abiding gun owners to purchase and possess firearms. In this study, we examined gun owners’ and non-gun owners’ attitudes about four policies that are consistent with this underlying principle: universal background checks, red flag laws, a permit to purchase or possess a firearm and permits for concealed carry. If crafted properly, each of these laws can play a major role in keeping guns out of the hands of people at a high risk for violence, while at the same time interfering minimally with gun owners’ ability to exercise their constitutional rights. As the combination of the survey and focus group results found, keeping guns out of the hands of those with an increased risk of committing a violent crime is supported by both sides and at the core of policies discussed here.

There are several ways that these laws can be crafted so as to not inconvenience gun owners while not sacrificing public safety. For example, we found that gun owners are concerned by the fact that under certain universal background check laws, it is a felony for them to lend a gun to family member for a hunting trip without going through a background check at an FFL. One way to remedy this, while at the same time strengthening a state’s ability to keep guns out of the hands of violent offenders, is to require a state permit to purchase or possess a gun. Under such a system, point-of-purchase background checks would not be needed: Instead, the seller or lender would have to validate that the purchaser or borrower possesses a currently valid, state-issued firearm permit. Thus, law-abiding gun owners would no longer have to go to an FFL when lending a gun to a family member, as long as the family member possesses a valid firearm permit, which documents that the state deems this individual legally qualified to possess a firearm. The connection between background checks and gun owner concerns that such checks would result in a registry carries an important policy implication: A not insignificant number of gun owners who support universal background checks would be against them if a registry comes attached.

Keeping the costs of permits low as well as including a firearm safety course will address additional gun owner concerns. We recognize that keeping costs low for firearm permits could pose a challenge if a safety training course is included. Further research is needed to determine how important low permit cost is to gun owners relative to the importance of having a safety training requirement. Since firearm safety training courses would be analogous to drivers' education classes, but shorter in duration, economic analysis should be performed to determine costs to run already existing programs in order to forecast approximate costs of such firearm courses. The entirety of the cost to operate safety courses also does not need to be absorbed by permit applicants. Congress could address this by providing a financial incentive intended to offset permit costs for states that enact permit requirements.

Firearm owners whose support for permits to purchase any firearm are contingent upon inclusion of a mandatory safety training course may view the training element as the reason a permit requirement would exist. While permits actually function to both rule out a criminal history and to ensure safety training, some firearm owners may view the requirement for safety training as a key reason to require a firearm permit in the first place.

For red flag laws, gun owner concerns could be addressed by including provisions that penalize anyone who makes a false and vindictive request for a protection order. This would not interfere with the effectiveness of red flag laws, but, according to our research findings, would ease the concerns of a huge number of gun owners. An additional red flag law provision could be to guarantee the expeditious return of firearms to the gun owner when the protection order expires. This in no way compromises public safety, but it would address gun owners concerns about timely restoration of a constitutional right.

## Limitations

This paper has some major limitations. First, non-gun owners were not surveyed. However, we conducted post-survey focus groups and interviews with this group in order to confirm the identified common ground between them and gun owners.

Next, social desirability bias may have been present among survey respondents as well as focus group and interview participants, despite their knowledge of their anonymous participation. In the survey we did not formally test how much a given provision changed survey responses (such as by randomly assigning survey items, some with a given policy provision and some without), which could have helped test whether a provision’s inclusion or absence would cause a person’s support for a policy to change. As above, we conducted post-survey focus groups and interviews with gun owners to confirm the importance of each provision’s importance to gun owners, albeit among a much smaller sample. Because of this potential bias, the results of this study should be interpreted with caution; it may appear as though a particular provision is more critical to garner support than is actually the case. In addition, the survey questions themselves may have inherent limitations, such as not including an answer choice in a multiple-choice list that best reflects respondents’ opinion to a given question, and failure to include pertinent policies in the questions themselves.

Certain gun reform policy topics were not included in the survey, such as increasing the age of sale of all firearms to 21, restricting the carrying of firearms in public areas, open carry restrictions, policies that strengthen penalties for straw purchasers, a possible code of conduct for FFLs and laws regulating the illegal trafficking of firearms. However, we did include all major laws for which previous surveys have found majority support among gun owners. Since our goal was to identify legal provisions supported by gun owners, we did not want to waste survey time inquiring about a large number of policies that we knew from previous data would not be supported by gun owners.

## Conclusion

Despite this paper’s limitations, it provides valuable information to the gun safety policy debate. This research informs the gun safety policy community about both gun owners' principles behind their views on gun safety policy, and their opinions on policy provisions that drive their support or opposition to firearm policy, specifically universal background checks, red flag laws, permits to purchase or possess a firearm and concealed carry permits. This research could help gun violence prevention advocates and policy makers create an effective package of gun policies that does not alienate gun owners or non-gun owners and that also does not compromise public safety. This research demonstrates that there is common ground between gun owners and non-gun owners in the principles underlying gun safety policy, and that this common ground is broad enough so that an effective, mutually agreed upon gun safety policy is possible. These findings call into question the assumption that the wide polarization on the gun issue precludes the development and enactment of effective and widely supported gun safety policies.

## Supplementary Information

**Additional file 1.** Survey instrument.

## Data Availability

Data will be shared with researchers upon request.

## Abbreviations

CDC: Centers for Disease Control and Prevention
NRA: National Rifle Association
NICS: National Instant Criminal Background Check System
NORC: NORC at the University of Chicago
FFL: Federal Firearms License
