# Supplementary material for: Gun owners’ assessment of gun safety policy: their underlying principles and detailed opinions
Source: Inj Epidemiol. 2023 Apr 17;10:21. doi: 10.1186/s40621-023-00430-z (PMC10111648; doi:10.1186/s40621-023-00430-z)
Supplement: Supplementary file 1 — Additional file 1. Survey instrument. [file 40621_2023_430_MOESM1_ESM.docx]

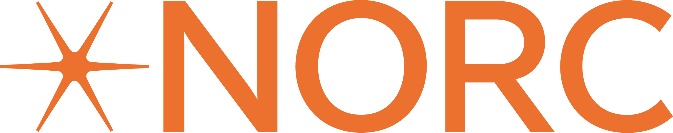

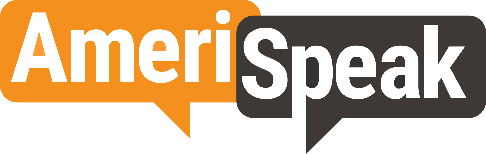


**Survey Questions***

**Gun Safety Common Ground Study 2022**

Conducted for: Tufts University

Conducted by: NORC at the University of Chicago

Sample Source: AmeriSpeak Probability-Based Panel

Sampled Population: US gun owners age 18+

Date Fielded: May 10-18, 2022

WINTRO_1.

Thank you for agreeing to participate in our new AmeriSpeak survey!

This survey is about violence and policy.

As always, your answers are confidential.

INTRO.

You are being asked to volunteer for a research study. This study is being run by the Tufts University School of Medicine. We are doing this study to understand your opinions about gun laws. We want to find out the possible benefits that gun laws may provide to gun owners. We also want to find out the details of gun policies that gun owners do not support. If you agree to participate, we will ask you to complete an internet survey. The survey will take no more than 25 minutes.

The researchers at Tufts University will not have access to your name or any personal information. Before the survey responses are shared with the researchers, all names and other personal information will be removed. Therefore, the researchers will not be able to link responses to any individual. This will ensure that your responses remain anonymous. There are no individual benefits to you for participating in this survey. The alternative to participation is choosing not to participate in the survey. This study has been reviewed by the Tufts Social Behavioral & Educational Research Institutional Review Board. This is a group that reviews all research projects that include human subjects.

GUNOWN.

Do you currently own one or more guns?

RESPONSE OPTIONS:

1. Yes
2. No

[IF GUNOWN=2, 77,98,99, TERMINATE]

Q1.

Please indicate your level of agreement or disagreement with the following statements about the reasons why you own firearms?

GRID ITEMS, RANDOMIZE:

A. I own firearms to manage pests on my property

B. I own firearms to protect my family

C. I own firearms to protect my community

D. I own firearms to exercise my constitutional rights

E. I own firearms because I like collecting them

F. I own firearms because it is a tradition in my family

G. I own firearms for my own protection

H. I own firearms because I enjoy hunting

I. I own firearms because I enjoy shooting for sport or competition

J. I own firearms because I use them for my job

RESPONSE OPTIONS:

1. Strongly Agree

2. Agree

3. Neither Agree nor Disagree

4. Disagree

5. Strongly Disagree

Q2.

What would you say is the primary reason that you own firearms, if you could choose only one reason?

RESPONSE OPTIONS, RANDOMIZE:

1. I own firearms to manage pests on my property

2. I own firearms to protect my family

3. I own firearms to protect my community

4. I own firearms to exercise my constitutional rights

5. I own firearms because I like collecting them

6. I own firearms because it is a tradition in my family

7. I own firearms for my own protection

8. I own firearms because I enjoy hunting

9. I own firearms because I enjoy shooting for sport or competition

10. I own firearms because I use them for my job

FIREARM_OWN.

The following questions are about firearms that you own. The researchers are asking these questions solely because they want to understand what types and models of firearms are commonly used by law-abiding gun owners for legal purposes. Your name and other identifying information will not be disclosed to the researchers.

Q3.

How many firearms do you personally own of each type below?

GRID ITEMS:

A. Pistols

B. Revolvers

C. Rifles

D. Shotguns

RESPONSE OPTIONS:

1. None (0)

2. 1

3. 2

4. 3

5. 4

6. 5 or more

99. Prefer not to answer

Q4.

Do you own any modern sporting rifles, such as AR15s (these are sometimes referred to as ‘assault weapons’ or ‘military-style semiautomatic rifles’)?

RESPONSE OPTIONS:

1. Yes

2. No

99. Prefer not to answer

Q5.

Please indicate your level of support or opposition to the following policies.

GRID ITEMS:

A. Prohibiting a person subject to a domestic violence restraining order from having a gun for the duration of the order

B. Prohibiting a person convicted of a crime of domestic violence from having a gun

C. Requiring background checks for every gun sale, including all private sales and at gun shows (universal background checks)

D. Temporarily confiscating firearms from people deemed by a judge to be a risk to themselves or others (red flag laws)

E. Giving law enforcement officers discretion in whether or not to approve a concealed carry permit application

F. Requiring a permit to purchase or possess any firearm

G. Requiring a permit to purchase or possess any handgun

H. Creating a federal database to track gun sales

I. Banning ammunition magazines that hold more than 10 rounds

J. Requiring by law that a person lock up the guns in their home when not in use

K. Requiring a permit to carry a concealed handgun

L. Requiring background checks to obtain a concealed carry permit

M. Requiring a firearm safety course before the purchase of a firearm

N. Requiring a firearm safety course before being issued a concealed carry permit

O. Banning military-style semi-automatic firearms (such as AR15s)

P. Allowing people convicted of non-violent felonies to retain their gun rights

RESPONSE OPTIONS:

1. Strongly support

2. Support

3. Neutral

4. Oppose

5. Strongly oppose

Q6.

Please indicate your level of agreement or disagreement with the following gun policy principles.

GRID ITEMS, RANDOMIZE:

A. People who have been convicted of a violent crime should not be able to purchase or possess a gun.

B. People who have been convicted of two or more drunk driving incidents should not be allowed to purchase or possess a gun.

C. One of the primary goals of gun laws should be to keep guns out of the hands of people who are at high risk of violence while allowing law-abiding citizens to obtain guns.

D. One of the primary goals of gun laws should be to develop criteria that keep guns out of the hands of people who are at high risk for violence, while not preventing access to guns by law-abiding citizens.

E. Owning a gun for self-defense is a fundamental Constitutional right.

F. Revising gun policies offers the opportunity to simplify the requirements for gun ownership, streamlining the process and making it much simpler and more efficient.

G. Laws that prohibit all people with mental health problems from possessing a gun are too broad and prevent many people who really are not a risk from owning a gun for self-defense.

H. The real risk for violence is a history of violence, so laws should be simplified so that the only people prohibited from owning a gun are those with a history of violence (i.e., a conviction for a violent crime).

I. Restrictions on the purchase and possession of guns can be imposed without necessarily taking away the right of law-abiding people to own guns.

J. I want to help find a way to reduce gun deaths and injuries.

K. The laws which are most essential to protect the public are those which state the criteria that either allow or disqualify a person from gun ownership.

L. I am concerned about the increase in gun-related homicides and gun crimes in cities.

M. I am concerned about the frequency of mass shootings.

N. I am concerned about the frequency of school shootings.

O. I am concerned about the issue of stolen guns.

P. Purchasing a gun should be a private transaction. No permanent record should be kept with identifying information about the purchaser, such as a registry of gun purchasers.

Q. Gun purchase transactions should record the purchaser’s name and background check result but not the number or type of guns that are purchased.

R. We don’t need any new gun laws. We just need to enforce the laws that already exist.

S. Gun laws are ineffective in reducing firearm violence.

T. Gun owners should be required to report the loss or theft of any of their firearms.

U. The right to own a gun is absolute and cannot be altered in any way.

V. Like free speech, the right to own a gun is Constitutionally protected, but there are exceptions (e.g., a person has free speech, but cannot yell “Fire” in a crowded theater; similarly, a person has the right to own a gun, but can lose that right under certain circumstances).

W. Ghost guns represent a major threat to public safety.

X. Implementing universal background checks for all gun sales, including those from private sellers, would likely result in the formation of a gun registry.

Y. I would support a universal background check law, even if it resulted in the creation of a gun registry.

Z. I am opposed to a gun registry that contains the name of individuals who own guns and the types/models of guns that they own.

AA. I am not opposed to a registry of gun transactions as long as it does not record the number and type/model of guns purchased or sold.

AB. I think there should be fines for gun owners who do not store their guns securely and a minor in that household accesses a gun.

AC. Obtaining a concealed carry permit should come with perks such as waiving the waiting period for gun purchases and not requiring additional gun safety testing.

AD. There should be no permit required to purchase a gun but there should be a permit required for concealed carry.

AE. Private gun sellers should be given privacy-protected access to the FBI’s National Instant Background Check System (NICS) database so that they can conduct a background check without having to go to a federally licensed gun dealer.

AF. Red flag laws should contain a provision that punishes individuals for making a dishonest accusation that a person is a danger to themselves or others.

AG. Red flag laws should contain a provision that requires a timely court hearing at which the subject can appear and present evidence before a gun is taken away for an extended period of time.

AH. If training is required before purchase of a firearm, the costs must be reasonable and there must be subsidies for people who cannot afford the cost.

AI. Taking a firearm safety course should come with perks such as waiving the waiting period for buying a gun and making it easier to obtain a concealed carry permit.

AJ. Gun owners should not be required by law to store their guns unloaded and locked because it might interfere with their ability to access their gun if needed for self-defense.

AK. Guns should be required to have a fingerprint recognition device so that they can only be accessed by the original owner.

RESPONSE OPTIONS:

1. Strongly agree

2. Agree

3. Neutral

4. Disagree

5. Strongly disagree

Q7_1.

Please indicate your level of agreement or disagreement with the following statements about your support for specific gun policies.

Universal Background Check Laws: A universal background check law requires a background check for every gun purchase, even from a private seller.

GRID ITEMS:

A. I would only support a universal background check law if it included an exemption to allow the sale and transfer of guns to a family member without a background check.

B. I would only support a universal background check law that did NOT exempt sales to family members if private sellers were given privacy-protected access to the NICS database, whereby a green light or red light would appear to indicate whether the person is eligible.

C. I would only support a universal background check law if it provided a way to sell or transfer firearms to family members without having to go through a federally licensed dealer (FFL).

D. I would only support a universal background check law if it did not result in the creation of a firearm registry (i.e., if a permanent record was kept of the name of the buyer and the type/model of guns purchased).

E. I would only support a universal background check law if it required a timely response from NICS check system, such as within 72 hours.

F. I would not support a universal background check law under any conditions including those listed above.

G. I would support a universal background check law even if it didn’t contain any of the provisions listed above.

RESPONSE OPTIONS:

1. Strongly agree

2. Agree

3. Neutral

4. Disagree

5. Strongly disagree

Q7_2.

Please indicate your level of agreement or disagreement with the following statements about your support for specific gun policies.

Red Flag Laws: A red flag law allows law enforcement officials to temporarily remove a firearm from someone who is deemed by a judge to be a danger to themselves or others.

GRID ITEMS:

A. I would only support a red flag law if it included a provision stating that the firearm could only be confiscated for an extended period time after a timely due process hearing in front of a judge, at which point the subject of the potential order could be present and provide evidence.

B. I would only support a red flag law if it included a provision stating that the request to remove the firearm from the person could only be made by a law enforcement officer, not a family member.

C. I would only support a red flag law if it included a fine for anyone who dishonestly uses the law to try to get firearms taken away from another person (for example, the person filing the complaint is just trying to get back at the gun owner for some reason).

D. I would only support a red flag law if it included a protocol for expeditious and inexpensive restoration of Second Amendment rights if the accusation proves to be unfounded or when the person is deemed to no longer represent a threat.

E. I would only support a red flag law if it included provisions for the expeditious return of the accused person’s firearms once the order is lifted and the accused person’s rights are restored.

F. I would only support a red flag law if it allowed the transfer of confiscated firearms to a designated friend or family member for safekeeping, instead of being stored by law enforcement officials.

G. I would not support a red flag law under any conditions including those listed above.

H. I would support a red flag law even if it didn’t contain any of the provisions listed above.

RESPONSE OPTIONS:

1. Strongly agree

2. Agree

3. Neutral

4. Disagree

5. Strongly disagree

Q7_3.

Please indicate your level of agreement or disagreement with the following statements about your support for specific gun policies.

Permit to Purchase Laws: A permit to purchase law requires that an individual obtain a permit or license in order to purchase or possess a gun.

GRID ITEMS:

A. I would only support a permit to purchase law if it required live firearm shooting training to obtain the license or permit.

B. I would only support a permit to purchase law if it exempted gun owners who have a permit from the need for a background check when purchasing or borrowing a gun from a family member.

C. I would only support a permit to purchase law if it exempted gun owners who have a permit from the need for a background check when purchasing any new gun.

D. I would only support a permit to purchase law if it could not be used to create a registry of firearms owned by each gun owner (i.e., the name of the person getting the license would be linked to the number and type of firearms that they purchase).

E. I would only support a permit to purchase law if it included perks associated with the permit, such as waiving of the normal waiting period for purchase of a gun.

F. I would only support a permit to purchase law if the permit were available at low cost.

G. I would only support a permit to purchase law if it allowed the permitting process to be completed online.

H. I would not support a permit to purchase law under any conditions including those listed above.

I. I would support a permit to purchase law even if it didn’t contain any of the provisions listed above.

RESPONSE OPTIONS:

1. Strongly agree

2. Agree

3. Neutral

4. Disagree

5. Strongly disagree

Q7_4.

Please indicate your level of agreement or disagreement with the following statements about your support for specific gun policies.

Concealed Carry Permit Laws: A concealed carry permit law is one that requires a permit or license to be obtained in order to be allowed to carry a concealed handgun.

GRID ITEMS:

A. I would only support a concealed carry permit law if it required live firearm shooting training as part of the procedure to obtain the permit.

B. I would only support a concealed carry permit law if it could not be used to create a registry of firearms owned by each gun owner (i.e., the name of the person getting the license would be linked to the number of type of handguns that they own).

C. I would only support a concealed carry permit law if it was “shall issue,” meaning that law enforcement officials would have no discretion to deny a permit if the applicant met all the specified criteria, such as not having a history of a violent crime.

D. I would only support a concealed carry permit law if it included perks associated with the carry permit, such as waiving of the normal waiting period for purchase of a gun.

E. I would only support a concealed carry permit law if the permit was available at low cost.

F. I would only support a concealed carry permit law if it allowed the application procedure to be completed online.

G. I would not support a concealed carry permit law under any conditions including those listed above.

H. I would support a concealed carry permit law even if it didn’t contain any of the provisions listed above.

RESPONSE OPTIONS:

1. Strongly agree

2. Agree

3. Neutral

4. Disagree

5. Strongly disagree

Q7_5.

Please indicate your level of agreement or disagreement with the following statements about your support for specific gun policies.

Safe Gun Storage Laws: A safe gun storage law is one that requires that all guns in the home be stored securely and inaccessible to minors.

GRID ITEMS:

A. I would only support a safe gun storage law if it contained a provision stating that one option for secure storage is having a gun with fingerprint recognition (or another type of biometric lock) so that the gun can be accessed quickly, but only by its owner.

B. I would only support a safe gun storage law if it included multiple options to comply with the law, beyond simply storing guns unloaded and locked.

C. I would only support a safe gun storage law if it exempted gun owners if there are no minors present in the household.

D. I would not support a safe gun storage law under any conditions including those listed above.

E. I would support a safe gun storage law even if it didn’t contain any of the provisions listed above.

RESPONSE OPTIONS:

1. Strongly agree

2. Agree

3. Neutral

4. Disagree

5. Strongly disagree

Q8.

At which level of government (state vs. federal) do you think each of the following gun policies should be enacted?

GRID ITEMS:

1. Universal background checks

2. Red flag laws

3. Concealed carry permit laws

4. Licensing and permitting laws for purchase or possession of firearms

5. Safe gun storage laws

6. Laws specifying the requirements for owning a gun (e.g., history of violent crime)

RESPONSE OPTIONS:

1. Neither level – I don’t support this policy

2. Federal level only

3. State level only

4. Both Federal and State levels

Q9.

In which of the following ways do you believe that gun laws could be better enforced?

*Please select all that apply.*

RESPONSE OPTIONS:

1. I do not think that gun laws need better enforcement.

2. Improve reporting of criminal history to the NICS system so that it captures a greater percentage of relevant crimes.

3. Improve the NICS system itself so that it is more efficient and provides much quicker responses.

4. Improve the reporting of crimes to state or local law enforcement personnel so that they can identify people who are no longer legally allowed to possess a gun.

5. More strongly enforce laws that prohibit trafficking of guns and straw purchases.

6. Give law enforcement the authority to remove firearms from people who become legally ineligible to possess them.

7. Respond more quickly and definitively when someone who possesses a firearm is identified as a danger to themselves or others.

8. None of the above items would improve enforcement of gun laws.

Q10_1.

Please estimate what percentage of other gun owners in the United States support the following policy.

Prohibiting a person subject to a domestic violence restraining order from having a gun for the duration of the order

RESPONSE OPTIONS:

1. 0%

2. 10%

3. 20%

4. 30%

5. 40%

6. 50%

7. 60%

8. 70%

9. 80%

10. 90%

11. 100%

Q10_2.

Please estimate what percentage of other gun owners in the United States support the following policy.

Prohibiting a person convicted of a crime of domestic violence from having a gun

RESPONSE OPTIONS:

1. 0%

2. 10%

3. 20%

4. 30%

5. 40%

6. 50%

7. 60%

8. 70%

9. 80%

10. 90%

11. 100%

Q10_3.

Please estimate what percentage of other gun owners in the United States support the following policy.

Requiring background checks at gun shows and for all private sales (universal background checks)

RESPONSE OPTIONS:

1. 0%

2. 10%

3. 20%

4. 30%

5. 40%

6. 50%

7. 60%

8. 70%

9. 80%

10. 90%

11. 100%

Q10_4.

Please estimate what percentage of other gun owners in the United States support the following policy.

Prohibiting gun possession by people deemed to be a risk to themselves or others (red flag laws)

RESPONSE OPTIONS:

1. 0%

2. 10%

3. 20%

4. 30%

5. 40%

6. 50%

7. 60%

8. 70%

9. 80%

10. 90%

11. 100%

Q10_5.

Please estimate what percentage of other gun owners in the United States support the following policy.

Requiring a permit to purchase or possess any firearm (i.e., any pistol, revolver, rifle, or shotgun)

RESPONSE OPTIONS:

1. 0%

2. 10%

3. 20%

4. 30%

5. 40%

6. 50%

7. 60%

8. 70%

9. 80%

10. 90%

11. 100%

Q10_6.

Please estimate what percentage of other gun owners in the United States support the following policy.

Requiring a permit to purchase or possess any handgun (i.e., a pistol or revolver)

RESPONSE OPTIONS:

1. 0%

2. 10%

3. 20%

4. 30%

5. 40%

6. 50%

7. 60%

8. 70%

9. 80%

10. 90%

11. 100%

Q10_7.

Please estimate what percentage of other gun owners in the United States support the following policy.

Requiring a permit to carry a concealed handgun

RESPONSE OPTIONS:

1. 0%

2. 10%

3. 20%

4. 30%

5. 40%

6. 50%

7. 60%

8. 70%

9. 80%

10. 90%

11. 100%

Q11.

For how many years have you been a gun owner?

RESPONSE OPTIONS:

1. Less than one year

2. 1 to less than 3 years

3. 3 to less than 5 years

4. 5 to less than 10 years

5. 10 years and more

Q13.

Are you a member of the NRA?

RESPONSE OPTIONS:

1. Yes

2. No

99. Prefer not to answer

[SHOW IF Q13 = 2, 77, 98, 99]

Q15.

Would you join the NRA if their choice of messaging was more inclusive and less divisive?

RESPONSE OPTIONS:

1. Yes

2. No

3. Not Sure

4. I am already an NRA member

Q14.

To what extent do you support the NRA?

RESPONSE OPTIONS:

1. Not at all

2. A little bit

3. A fair amount

4. A lot

5. Completely

Q12.

Are you a member of any gun rights organization?

RESPONSE OPTIONS:

1. Yes

2. No

99. Prefer not to answer

**Demographic Profile:**

**Additional questions asked of panelists prior to this survey**

**and are included with the survey data**

| Variable | Values |
| --- | --- |
| Gender | 1 = Male |
|  | 2 = Female |
| Age | Age in years |
| Age (7 categories) | 1 = 18-24; 2 = 25-34; 3 = 35-44; 4 = 45-54; 5 = 55-64; 6 = 65-74; 7 = 75+ |
| Age (4 categories) | 1 = 18-29; 2 = 30-44; 3 = 45-59; 4 = 60+ |
| Education (5 categories) | 1 = Less than HS |
|  | 2 = HS graduate |
|  | 3 = Vocational/tech school/some college/associates |
|  | 4 = Bachelor’s degree |
|  | 5 = Post grad study/professional degree |
| Race/Ethnicity | 1 = White, Non-Hispanic |
|  | 2 = Black, Non-Hispanic |
|  | 3 = Other, Non-Hispanic |
|  | 4 = Hispanic |
|  | 5 = 2+ races, Non-Hispanic |
|  | 6 = Asian/Pacific Islander, Non-Hispanic |
| Housing Type | 1 = A one-family house detached from any other house |
|  | 2 = A one-family house attached to one or more houses |
|  | 3 = A building with 2 or more apartments |
|  | 4 = A mobile home or trailer |
|  | 5 = Boat, RV, van, etc. |
| Household Income (18 categories) | 1 = Less than $5,000 2 = $5,000 to $9,999 |
|  | 3 = $10,000 to $14,999 4 = $15,000 to $19,999 |
|  | 5 = $20,000 to $24,999 6 = $25,000 to $29,999 |
|  | 7 = $30,000 to $34,999 8 = $35,000 to $39,999 |
|  | 9 = $40,000 to $49,999 10 = $50,000 to $59,999 |
|  | 11 = $60,000 to $74,999 12 = $75,000 to $84,999 |
|  | 13 = $85,000 to $99,999 14 = $100,000 to $124,999 |
|  | 15 = $125,000 to $149,999 16 = $150,000 to $174,999 |
|  | 17 = $175,000 to $199,999 18 = $200,000 or more |
| Household Income (9 categories) | 1 = Less than $10,000 |
|  | 2 = $10,000 to $19,999 |
|  | 3 = $20,000 to $29,999 |
|  | 4 = $30,000 to $39,999 |
|  | 5 = $40,000 to $49,999 |
|  | 6 = $50,000 to $74,999 |
|  | 7 = $75,000 to $99,999 |
|  | 8 = $100,000 to $149,999 |
|  | 9 = $150,000 or more |
| Household Income (4 categories) | 1 = Less than $30,000 |
|  | 2 = $30,000 to $59,999 |
|  | 3 = $60,000 to $99,999 |
|  | 4 = $100,000 or more |
| Marital Status | 1 = Married |
|  | 2 = Widowed |
|  | 3 = Divorced |
|  | 4 = Separated |
|  | 5 = Never married |
|  | 6 = Living with partner |
| Metropolitan Statistical Area  Status | 0 = Non-Metro |
|  | 1 = Metro (as defined US OMB Core-Based Statistical Area) |
| Home Internet Access | 0 = No |
|  | 1 = Yes |
| Telephone Service | 1 = Landline telephone only |
|  | 2 = Have a landline, but mostly use cellphone |
|  | 3 = Have cellphone, but mostly use landline |
|  | 4 = Cellphone only |
|  | 5 = No telephone service |
| Ownership of Living Quarters | 1 = Owned or being bought by you or someone in your household |
|  | 2 = Rented for cash |
|  | 3 = Occupied without payment of cash rent |
| Region 4 (US Census) | 1 = Northeast |
|  | 2 = Midwest |
|  | 3 = South |
|  | 4 = West |
| Region 9 (US Census) | 1 = New England |
|  | 2 = Mid-Atlantic |
|  | 3 = East-North Central |
|  | 4 = West-North Central |
|  | 5 = South Atlantic |
|  | 6 = East-South Central |
|  | 7 = West-South Central |
|  | 8 = Mountain |
|  | 9 = Pacific |
| State | State of residence |
| Household Size | Total number of members in household |
| HH members, age 0-1 | Number of household members in age group |
| HH members, age 2-5 | Number of household members in age group |
| HH members, age 6-12 | Number of household members in age group |
| HH members, age 13-17 | Number of household members in age group |
| HH members, age 18+ | Number of household members in age group |
| Current Employment Status | 1 = Working - as a paid employee |
|  | 2 = Working - self-employed |
|  | 3 = Not working - on temporary layoff from a job |
|  | 4 = Not working - looking for work |
|  | 5 = Not working – retired |
|  | 6 = Not working – disabled |
|  | 7 = Not working – other |
| Political Party | 1 = Strong Democrat |
|  | 2 = Not so strong Democrat |
|  | 3 = Lean Democrat |
|  | 4 = Don't Lean/Independent/None |
|  | 5 = Lean Republican |
|  | 6 = Not so strong Republican |
|  | 7 = Strong Republican |
| Political Opinions | 1 = Very liberal |
|  | 2 = Somewhat liberal |
|  | 3 = Moderate |
|  | 4 = Somewhat conservative |
|  | 5 = Very conservative |
